# Galectins and Their Ligand Glycoconjugates in the Central Nervous System Under Physiological and Pathological Conditions

**DOI:** 10.3389/fnana.2021.767330

**Published:** 2021-10-15

**Authors:** Junko Nio-Kobayashi, Tetsuya Itabashi

**Affiliations:** Laboratory of Histology and Cytology, Faculty of Medicine and Graduate School of Medicine, Hokkaido University, Sapporo, Japan

**Keywords:** galectin, glycan, neurodegenerative disease, Mgat5/GnTV, keratan sulfate, post-translational modification, glucose metabolism

## Abstract

Galectins are β-galactoside-binding lectins consisting of 15 members in mammals. Galectin-1,-3,-4,-8, and -9 are predominantly expressed in the central nervous system (CNS) and regulate various physiological and pathological events. This review summarizes the current knowledge of the cellular expression and role of galectins in the CNS, and discusses their functions in neurite outgrowth, myelination, and neural stem/progenitor cell niches, as well as in ischemic/hypoxic/traumatic injuries and neurodegenerative diseases such as multiple sclerosis. Galectins are expressed in both neurons and glial cells. Galectin-1 is mainly expressed in motoneurons, whereas galectin-3-positive neurons are broadly distributed throughout the brain, especially in the hypothalamus, indicating its function in the regulation of homeostasis, stress response, and the endocrine/autonomic system. Astrocytes predominantly contain galectin-1, and galectin-3 and−9 are upregulated along with its activation. Activated, but not resting, microglia contain galectin-3, supporting its phagocytic activity. Galectin-1,−3, and -4 are characteristically expressed during oligodendrocyte differentiation. Galectin-3 from microglia promotes oligodendrocyte differentiation and myelination, while galectin-1 and axonal galectin-4 suppress its differentiation and myelination. Galectin-1- and- 3-positive cells are involved in neural stem cell niche formation in the subventricular zone and hippocampal dentate gyrus, and the migration of newly generated neurons and glial cells to the olfactory bulb or damaged lesions. In neurodegenerative diseases, galectin-1,-8, and -9 have neuroprotective and anti-inflammatory activities. Galectin-3 facilitates pro-inflammatory action; however, it also plays an important role during the recovery period. Several ligand glycoconjugates have been identified so far such as laminin, integrins, neural cell adhesion molecule L1, sulfatide, neuropilin-1/plexinA4 receptor complex, triggering receptor on myeloid cells 2, and T cell immunoglobulin and mucin domain. *N*-glycan branching on lymphocytes and oligodendroglial progenitors mediated by β1,6-*N*-acetylglucosaminyltransferase V (Mgat5/GnTV) influences galectin-binding, modulating inflammatory responses and remyelination in neurodegenerative diseases. De-sulfated galactosaminoglycans such as keratan sulfate are potential ligands for galectins, especially galectin-3, regulating neural regeneration. Galectins have multitudinous functions depending on cell type and context as well as post-translational modifications, including oxidization, phosphorylation, S-nitrosylation, and cleavage, but there should be certain rules in the expression patterns of galectins and their ligand glycoconjugates, possibly related to glucose metabolism in cells.

## Introduction

**Figure 1:**
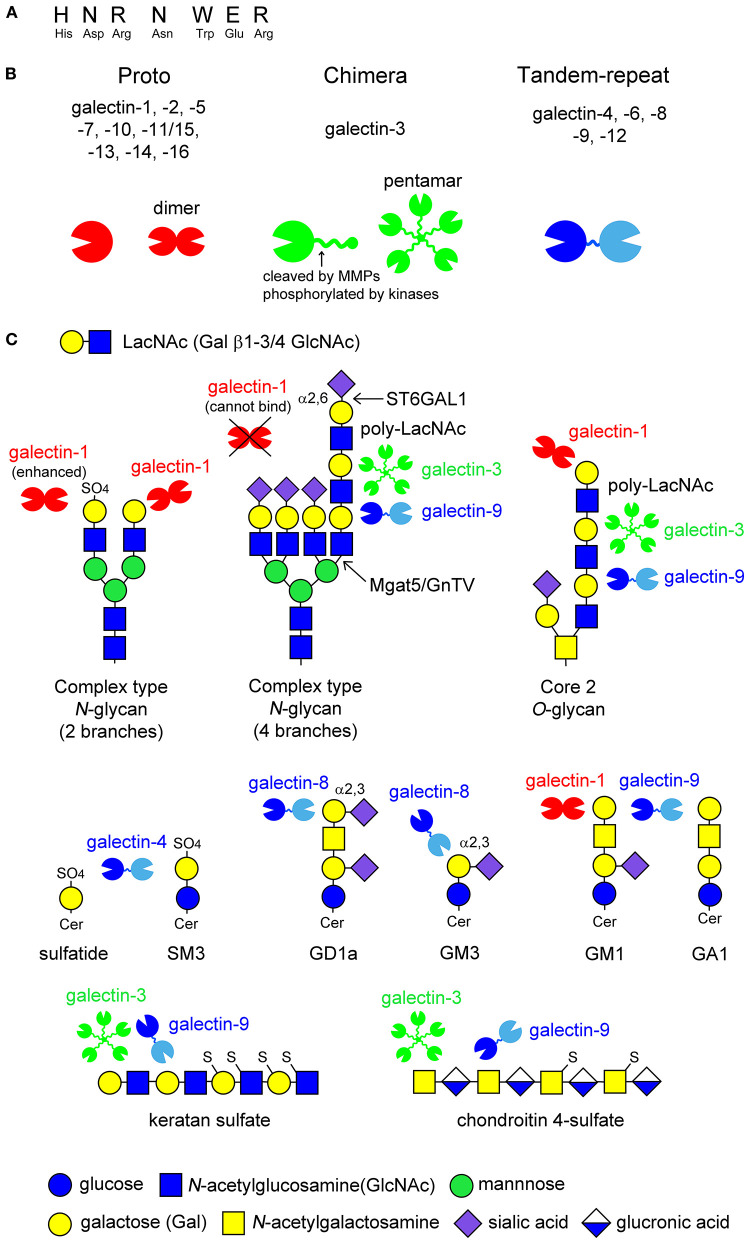
Schematic image of the structure of galectins and the ligand glycans. Galectins contain seven conserved amino acids sequence **(A)**. Fifteen members of galectins are classified into three types: proto type, chimera type, and tandem-repeat type. Proto type galectins have one carbohydrate recognition domain (CRD) and exist as dimers, whereas chimera type galectin-3 consists of *C*-terminal CRD and non-lectin *N*-terminal peptide, which can be phosphorylated by kinases or cleaved by proteinases, such as matrix metalloproteinases (MMPs) (*arrow* in **B**). *N*-terminal peptide is involved in self-association to form pentamer. Tandem-repeat type galectins possess two CRDs with different glycan-binding properties, that are linked by short peptide **(B)**. Known glycan structures for each galectin are shown in **(C)**. Galectins recognize *N*-acetyllactosamine (LacNAc) unit consisting of galactose (Gal) β1-3/4 linked to *N*-acetylglucosamine (GlcNAc). The binding of galectins to LacNAc is enhanced by 3-*O*-sulfation (galectin-1) or the formation of poly-LacNAc units (galectin-3 and -9), while α2,6-sialylation on terminal Gal blocks galectin-1-binding. Galectin-4 binds to 3-*O*-sulfated glycolipids such as sulfatide and SM3. Galectin-8 and -9 have affinity to GD1a and GM3, and GM1 and GA1, respectively. The binding of galectin-1 to ganglioside GM1 seems to depend on its clustering on lipid raft. De-sulfated keratan sulfate and chondroitin 4-sulfate are potential ligands for galectin-3 and -9.

Galectins are β-galactoside-binding animal lectins, consisting of 15 members in mammals. Galectins possess carbohydrate recognition domains (CRDs) consisting of approximately 130 amino acids, which contain seven conserved amino acids sequence required for carbohydrate recognition (Figure 1A). Galectins are structurally classified into three groups (Figure 1B) (Hirabayashi and Kasai, 1993): proto type (galectin-1,-2,-5,-7,-10,-11/15,-13,-14,-16), chimera type (galectin-3), and tandem-repeat type (galectin-4,-6,-8,-9,-12). Proto type galectins have one CRD and exist as dimers, whereas chimera type galectin-3 consists of one CRD and a non-lectin *N*-terminal peptide, inducing galectin-3 self-association to form a pentamer. Tandem-repeat type galectins contain two CRDs with different glycan-binding affinities (Figure 1B).

Galectins recognize β-linked galactose (Gal), such as *N*-acetyllactosamine (LacNAc), consisting of Gal β1-3/4 linked to *N*-acetylglucosamine (GlcNAc), in cell surface glycoproteins and glycolipids, as well as proteoglycans in the extracellular matrix (Figure 1C). The glycan-binding affinity for each galectin differs depending on the glycan structure and modification of the Gal residue by fucosylation, sialylation, and sulfation (Hirabayashi et al., 2002; Rabinovich and Toscano, 2009). Generally, the binding affinity of galectins is enhanced by increased branching of complex type *N*-glycans or a repeated LacNAc motif. For example, galectin-1 binds to the LacNAc motif on complex type *N*-glycans, whereas galectin-3 and -9 have a high affinity for multiple LacNAc units formed on the fourth branch of complex type *N*-glycans and core 2 type *O*-glycans (Figure 1C). Modification at the C6 position of Gal, such as α2,6-sialylation catalyzed by β-galactoside α2,6-sialyltransferase 1 (ST6GAL1), inhibits the binding of galectins, such as galectin-1, although galectin-3 and -9 sustains their binding affinity (Figure 1C). In contrast, 3-*O*-sulfation enhances the galectin-1 binding to LacNAc (Figure 1C). Galectin-4 binds to 3-*O*-sulfated galactose-carrying glycolipids such as sulfatide and SM3. Galectin-8 and -9 have a high affinity for GD1a and GM3, GM1 and GA1, respectively (Figure 1C). Galectin-1 is also reported to bind to ganglioside GM1; however, its binding seems to depend on its clustering in the membrane microdomains (Figure 1C). De-sulfated keratan sulfate and chondroitin 4-sulfate are also potential ligands for galectin-3 and -9 (Figure 1C).

Since galectins can bind to multiple glycoconjugate ligands in the body as well as microorganisms, they are involved in various physiological and pathological events, including cell proliferation, differentiation, apoptosis, autophagy, and signal transduction regulating development, immune responses, and cancer progression (Liu and Rabinovich, 2005; Yang et al., 2008; Cummings et al., 2017). Galectins exist in the cytoplasm, sensing membrane damage in the cell by the exposure of glycan chains of intraluminal glycans (Thurston et al., 2012; Jia et al., 2020). They are also secreted extracellularly *via* unknown mechanisms as leaderless secretory proteins because they lack a recognizable signal sequence for their transport into the classical endoplasmic reticulum-Golgi cargo trafficking machinery.

Galectins are broadly distributed in the mammalian body in tissue- and cell-specific manners (Nio-Kobayashi, 2017). Galectins are abundant in the gastrointestinal tract, immune system, and nervous system. This review summarizes the current knowledge of the expression patterns of each galectin in the central nervous system (CNS), and their functions in the regulation of neuronal and glial cell properties, as well as in the pathogenesis of neurodegenerative diseases.

## Galectins in the Neuron in the CNS

**Figure 2:**
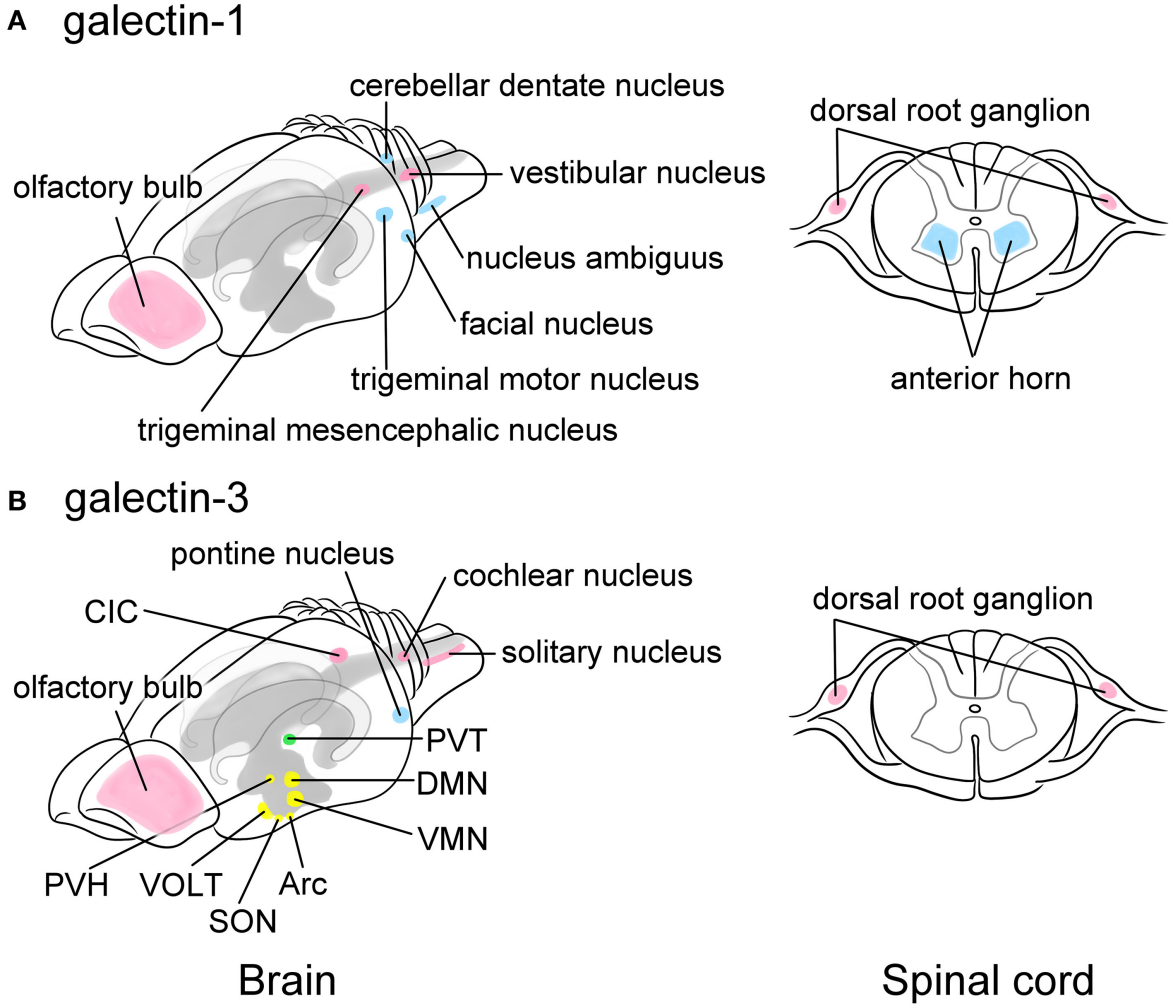
Schematic images showing the predominant localization of galectin-1 **(A)** and -3 **(B)** in neurons of the adult rat brain (left) and the spinal cord (right). Galectin-1 is predominantly localized to motoneurons or neurons controlling voluntary movements such as the facial nucleus, the trigeminal motor nucleus, the nucleus ambiguus, and the cerebellar dentate nucleus in the brain stem as well as the anterior horn of the spinal cord (blue in **A**). Certain sensory neurons, including the trigeminal mesencephalic nucleus and the vestibular nucleus in the brain and the neurons in the dorsal root ganglion (DRG) also contain galectin-1 (pink in **A**). Galectin-3 is abundant in the arcuate nucleus (Arc) and the dorsomedial nucleus (DMN), and moderately expressed in the ventromedial nucleus (VMN), the supraoptic nucleus (SON), and the paraventricular nucleus (PVH) in the hypothalamus that play central role in the regulation of appetite control, endocrine/autonomic system, and stress response (yellow in **B**). Galectin-3 is also present in the vascular organ of the lamina terminalis (VOLT), one of the sensory circumventricular organs (yellow in **B**). The paraventricular nucleus in the thalamus (PVT) related to the emotion also highly expresses galectin-3 (green in **B**). Other nuclei such as the central nucleus of inferior colliculus (CIC), the cochlear nucleus, and the solitary nucleus also express galectin-3 (pink in **B**), suggesting its role in the regulation of taste and hearing sensing. Similar to galectin-1, the primary sensory neurons in the DRG express galectin-3 (pink in **B**). The pontine nucleus regulating voluntary movements also contains abundant galectin-3 (blue in **B**). Olfactory bulb expresses both galectins (pink in **A** and **B**). Motoneurons and neurons involved in movement are represented in blue, sensory-related neurons are in pink, and the neurons regulating endocrine/autonomic functions are indicated in yellow, and nucleus related to higher brain function is labeled in green. The cerebral ventricles are indicated in gray and the hippocampus is bordered in light gray. This figure is created based on the *in situ* hybridization data by Hynes et al. (1990) for galectin-1 and immunohistochemical data by Yoo et al. (2017) for galectin-3. For detailed localization of galectins in the rat brain, please refer their manuscripts. For data of the mouse and human brain, please visit Allen Brain Map (https://portal.brain-map.org).

Galectin-1,-3,-4,-8, and -9 are expressed in neurons in the CNS. Hynes et al. (1990) analyzed galectin-1 expression in embryonic and adult rat nervous systems using RNA blot and *in situ* hybridization analyses. Galectin-1 expression is found in primary sensory neurons in the dorsal root ganglion (DRG) as well as in motoneurons in the anterior horn of the spinal cord and the motor nuclei in the brain stem, including the facial motor nucleus, the trigeminal motor nucleus, and the nucleus ambiguus. In addition, the cerebellar dentate nucleus responsible for the planning, initiation, and control of voluntary movements, and sensory neurons, such as the trigeminal mesencephalic nucleus and the vestibular nuclei, also express galectin-1 (Figure 2A). Other brain regions, including the midbrain, cerebellum, and hypothalamus, contain a much lower expression of galectin-1 mRNA, whereas the cerebral cortex, hippocampus, and corpus striatum express barely detectable levels of galectin-1 mRNA. The galectin-1 mRNA expression is much higher in the DRG and spinal cord than in the brain and is detectable soon after neuronal differentiation (Hynes et al., 1990).

Although mRNA expression remains unclear in the rat brain, Yoo et al. (2017) immunohistochemically revealed that galectin-3 is broadly distributed in neurons of adult rat brain (Figure 2B), especially in the hypothalamus, which plays a central role in the regulation of endocrine/autonomic regulation, stress response, and appetite control. In the hypothalamus, the arcuate nucleus and the dorsomedial nucleus abundantly contain galectin-3. The supraoptic nucleus, the paraventricular nucleus, and the ventromedial nucleus in the hypothalamus also express moderate level of galectin-3. Notably, galectin-3 expression is found in the vascular organ of the lamina terminalis (VOLT), one of the sensory circumventricular organs (CVOs) (Figure 2B). The sensory CVOs, which lack the blood-brain barrier, consist of subfornical organs and area postrema in addition to VOLT, monitoring the conditions of both the blood and the cerebrospinal fluids, as well as various sensory stimulations such as light, smell, and noise. The paraventricular nucleus in the thalamus, which is involved in emotion-related behaviors, contains high level of galectin-3. Intense immunoreactivities for galectin-3 are found in the central nucleus of inferior colliculus and the cochlear nucleus, as well as in the solitary nucleus, suggesting its role in the regulation of auditory and taste sensation. The pontine nucleus involved in motor activity is also immunoreactive for galectin-3. In contrast, the subcortical nuclei responsible for controlling voluntary motor functions and the limbic system exhibited no galectin-3 immunoreactivity. Note that in the mouse brain galectin-3 mRNA is not likely expressed in neurons according to the data from Allen Brain Atlas (https://mouse.brain-map.org).

The galectin-4 expression in the rat brain was analyzed by western blot analysis (Stancic et al., 2012; Velasco et al., 2013). Galectin-4 is expressed in the rat brain during the early postnatal developmental stage, decreases on days 12–16 onwards, being consistent with the onset of myelination, and becomes undetectable in adults (Stancic et al., 2012). Galectin-4 is localized to the cerebral cortex, hippocampal formation, thalamus, and corpus callosum, where both neurons and oligodendrocytes are immunoreactive (Stancic et al., 2012; Velasco et al., 2013). Interestingly, galectin-4 displays a segmented distribution in axons of cultured mature neurons (Velasco et al., 2013), which is involved in the transport of axonal glycoproteins and promotion of axonal growth but prevention of myelination.

Galectin-8 is expressed in the thalamus, choroid plexus, and weakly in the hippocampus and cortex in mice. Galectin-8 is produced by primary cultured hippocampal neurons and protects them from damaging conditions such as nutrient deprivation, glutamate-induced excitotoxicity, hydrogen peroxide-induced oxidative stress, and amyloid β (Aβ) aggregation (Pardo et al., 2019). John and Mishra (2016) carried out mRNA transcriptomics of galectins using the Allen Brain Atlas in young adult mouse brains and microarray data of the human brain. Galectin-9 is broadly distributed in the mouse brain, including the cerebral cortex, olfactory bulb, basal ganglia, hippocampus, thalamus, hypothalamus, amygdala, cerebellum, and substantia nigra, whereas galectin-1 is almost ubiquitously expressed in the human brain. The distribution of galectin-8 is similar between mice and humans and is expressed in the limbic region related to learning, memory, and emotions, and the substantia nigra for motor movements. They also noted the expression of other galectins such as galectin-2,-7 and -12 in the mouse and human brain.

The olfactory bulb transmits smell information from the nose to the brain and receives information from the amygdala, neocortex, hippocampus, locus coeruleus and substantia nigra for odorant/chemical awareness, and predominantly express galectin-1 and -3 (Figure 2). Mahanthappa et al. (1994) first demonstrated the localization of galectin-1 in non-neuronal cells in the olfactory bulb of developing mice, suggesting its function in promoting olfactory axon fasciculation. Prof. Brian Key et al. have extensively studied the expression and function of galectin-1 and -3 in the olfactory system (Puche and Key, 1995; Puche et al., 1996; Tenne-Brown et al., 1998; St John and Key, 1999; Storan et al., 2004). In mice lacking the *Lgals1* gene, which encodes galectin-1, a subset of primary sensory olfactory axons fails to project to their correct target sites in the caudal olfactory bulb (Puche et al., 1996). Galectin-1 is also expressed in periglomerular cells and granule cells in the ventromedial region of adult mice (St John and Key, 1999). In addition, galectin-4,-7, and -8 are expressed by primary olfactory axons as they grow from the nasal cavity to the olfactory bulb (Storan et al., 2004). Galectins in the olfactory system have neurite outgrowth-promoting activity and play a role in neuronal pathfinding through the rostral migratory stream from the neural stem/progenitor cell niche, as described below.

## Galectins in Glial Cells in the CNS

**Figure 3:**
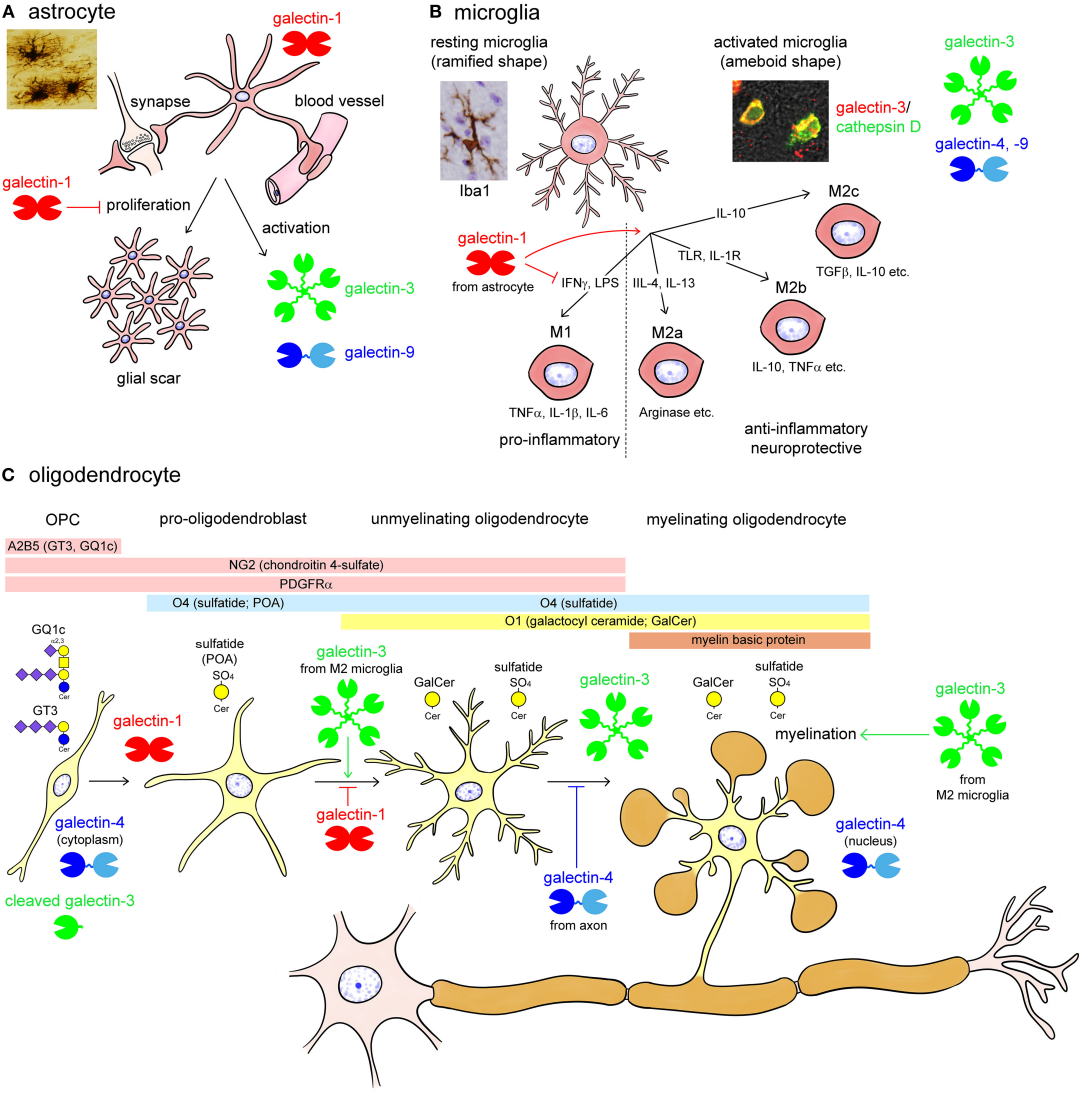
Summary images of galectin expression in glial cells (**A**; astrocytes, **B**; microglia, and **C**; oligodendrocytes) in the central nervous system (CNS). Astrocytes are supporting cells and surround the synapse and the blood vessels forming the blood-brain barrier in the CNS **(A)**. Astrocytes predominantly contain galectin-1 and activated astrocytes express galectin-3 and -9 **(A)**. Astrocytes proliferate in response to brain injury, forming a glial scar. Galectin-1 inhibits the proliferation and promotes differentiation of astrocytes, protecting neural function **(A)**. Microglia is responsible for controlling neural inflammation **(B)**. Resting microglia with elongated fine processes, so-called ramified-shaped microglia, activated by various stimuli including interferon γ (IFNγ), lipopolysaccharides (LPS), interleukin (IL)-4, IL-13, toll-like receptor (TLR) and IL-1 receptor stimulation, and IL-10 into pro-inflammatory M1 phenotype and anti-inflammatory, neuroprotective M2 (M2a, M2b, and M2c) phenotypes with ameboid shape **(B)**. Activated microglia abundantly contain galectin-3, and also express galectin-4 and -9. On the contrary, galectin-1 de-activates M1 type microglia, facilitating the shift from M1 to M2 phenotype **(B)**. Oligodendrocytes are myelinating cells in the CNS and are differentiated from oligodendrocyte precursor cells (OPC) reactive for monoclonal antibody (mAb) A2B5, which recognizes c-series gangliosides such as GT3 and GQ1c **(C)**. OPCs are also immunoreactive for chondroitin 4-sulfate-recognizing NG2 antibody and express platelet-derived growth factor receptor α (PDGFRα) **(C)**. Pro-oligodendroblasts contain sulfatide recognized by mAb O4 in addition to the OPC markers, NG2 and PDGFRα, and differentiated into oligodendrocytes, which contain galactosyl ceramide (GalCer) reactive for mAb O1. O4 recognizes sulfatide with short-chain fatty acids in pro-oligodendroblasts, called pro-oligodendroblast antigen (POA). Myelinating oligodendrocytes contain myelin basic protein forming myelin membrane around the axon. Galectin-1 is expressed in O1-negative OPC and pro-oligodendroblasts whereas galectin-3 is abundant in differentiated oligodendrocytes **(C)**. Galectin-4 localizes to the cytoplasm of OPC whereas in the nuclei of oligodendrocytes **(C)**. Galectin-1 and axonal galectin-4 inhibit the differentiation of oligodendrocytes; however, galectin-3, possibly from activated M2 type microglia, promotes oligodendrocyte differentiation and myelination **(C)**. Note that galectin-3 expressed in OPC is cleaved by a matrix metalloproteinase. TGFβ, transforming growth factor β; TNFα, tumor necrosis factor α.

Glial cells also express multiple galectins. Astrocytes are supporting cells in the CNS, surround the blood-brain barrier and synapse, and proliferate following brain injury to form a glial scar (Figure 3A). Astrocytes predominantly contain galectin-1, and activated astrocytes express galectin-3 and -9 (Sasaki et al., 2004; Yang et al., 2012; Steelman et al., 2013). Sasaki et al. (2004) reported that galectin-1 promoted astrocyte differentiation, but inhibited proliferation. Galectin-1 is remarkably upregulated in activated astrocytes around ischemic infarct, inhibiting astrocyte proliferation to downregulate astrogliosis (Qu et al., 2011). Similarly, galectin-1 suppresses microglial activation and protects against neural damage caused by inflammation (Starossom et al., 2012). The galectin-1 expression is also found in activated astrocytes and activated microglia in the injured rat spinal cord (Gaudet et al., 2015). Galectin-9 is not expressed in astrocytes under normal conditions, whereas its expression is increased by tumor necrosis factor α (TNFα) as well as interleukin (IL)-1β and interferon γ (IFNγ) stimulation in primary cultured astrocytes (Steelman et al., 2013).

Microglia are yolk sac-derived resident macrophages that play an important role in the regulation of immune responses in the CNS. Resting microglia show a ramified shape with elongated fine processes and disperse in the CNS (Figure 3B). Microglia are activated in response to CNS injury, and several phenotypes of activated microglia, also called ameboid-shaped microglia, have been reported so far: M1 type microglia produce pro-inflammatory cytokines such as TNFα, IL-1β, and IL-6. However, M2 (M2a, b, c) type microglia actively phagocytose cellular debris and secrete anti-inflammatory cytokines, including IL-10 and transforming growth factor β, as well as neurotropic molecules, such as insulin-like growth factor 1 (IGF1), promoting convergence of inflammation, synapse reconstitution, and remyelination (Figure 3B). Activated but not resting microglia contain abundant galectin-3. Galectin-3 is upregulated in both M1 and M2 type activated microglia (Figure 3B). Galectin-3 expression in microglia is enhanced by ischemic conditions or traumatic brain injury (Walther et al., 2000; Lalancette-Hébert et al., 2007; Yan et al., 2009; Venkatesan et al., 2010; Wesley et al., 2013; Yip et al., 2017; Rahimian et al., 2018; Wang et al., 2019). Treatment with trimethyltin, an organotin neurotoxicant, also induces galectin-3 expression in microglia and astrocytes in the hippocampal dentate gyrus (HDG) (Yang et al., 2012). Although galectin-3 has pro-inflammatory action, it also facilitates phagocytic activity and promotes neural recovery and post-ischemic tissue remodeling (Lalancette-Hébert et al., 2007). Galectin-9 is also reported to be increased in microglia following poly (I:C) stimulation (Steelman and Li, 2014). Although galectin-4 is virtually negligible in both microglia and astrocytes under normal conditions (Stancic et al., 2012), it is expressed in activated microglia in the brain and spinal cord of cuprizone-induced demyelination models and multiple sclerosis lesions (de Jong et al., 2018).

Oligodendrocytes are myelinating cells in the CNS equivalent to Schwann cells in the peripheral nervous system (PNS). Oligodendrocytes are produced from oligodendrocyte precursor cells (OPC), which are reactive for the monoclonal antibody (mAb) A2B5. This antibody recognizes c-series gangliosides such as GT3 and GQ1c (Farrer and Quarles, 1999; Saito et al., 2001). OPC are also immunoreactive for the chondroitin 4-sulfate-recognizing NG2 antibody, and express platelet-derived growth factor receptor α (PDGFRα). Pro-oligodendroblasts differentiated from OPC are immunoreactive for the mAb O4, which recognizes sulfatide, and differentiated into oligodendrocytes that contain galactosyl ceramide reactive for the mAb O1 (Sommer and Schachner, 1981; Bansal et al., 1992). Then, they are finally differentiated to myelinating oligodendrocytes, expressing myelin basic protein (MBP) to form myelin membranes around the axon (Figure 3C). Galectin-1 is expressed in A2B5/PDGFRα/O4-positive OPC and pro-oligodendroblasts but not in O1-positive oligodendrocytes in the primary culture of murine oligodendrocytes. In contrast, galectin-3 is upregulated during oligodendrocyte differentiation (Figure 3C) (Pasquini et al., 2011). Galectin-3 appears to be cleaved in OPC by matrix metalloproteinase 2 (MMP2), which results in a CRD peptide lacking the ability to form oligomers but having increased carbohydrate-binding activity (Figure 3C) (Ochieng et al., 1994; Gao et al., 2017). Both OPCs and mature oligodendrocytes contain galectin-4, with differential immunohistochemical localization: galectin-4 is present in the cytoplasm of OPC but accumulates in the nucleus of mature oligodendrocytes (Figure 3C) (Stancic et al., 2012). Extracellularly added galectin-3 promotes oligodendrocyte differentiation and myelination, whereas galectin-1 inhibits oligodendrocyte differentiation, suggesting that galectin-1 and -3 exert contrasting effects during the process of oligodendrocyte differentiation (Figure 3C) (Pasquini et al., 2011). Galectin-4 is released from neurons but not from oligodendrocytes, and inhibits myelination by binding to premyelinating oligodendrocytes, determining the timing of myelination (Figure 3C) (Stancic et al., 2012; Díez-Revuelta et al., 2017).

Galectin-1 and -3 are expressed in olfactory ensheathing cells; glial cells resemble Schwann cells in the PNS in ventromedial and lateral surfaces of the olfactory bulb in developing mice (Tenne-Brown et al., 1998; St John and Key, 1999). Ependymal cells in the subventricular zone (SVZ), where neural stem/progenitor cells are present, contain galectin-3 (Comte et al., 2011; Pasquini et al., 2011), and galectin-8 is expressed in the choroid plexus and secreted into the cerebrospinal fluid to protect neurons and suppress immune responses (Pardo et al., 2019).

## Galectins in Neurite Outgrowth and Myelination

**Figure 4:**
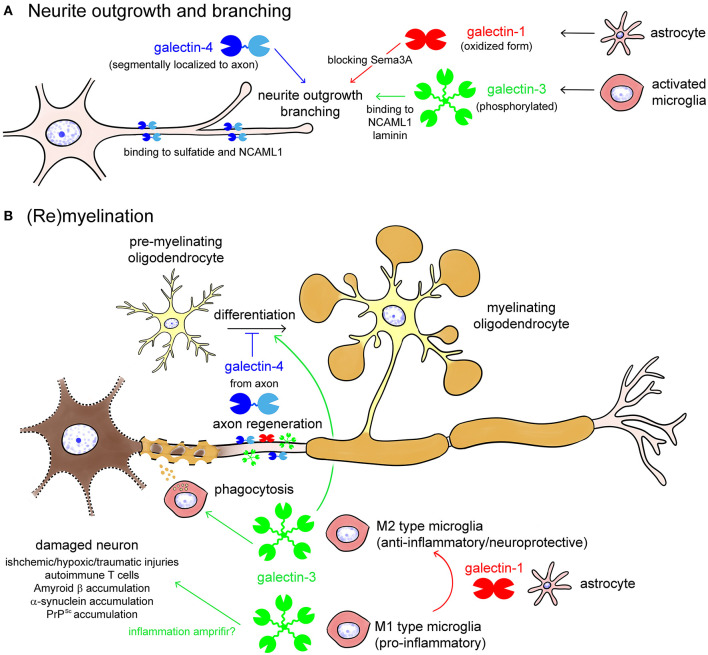
Galectins in neurite outgrowth and branching **(A)** as well as (re)myelination **(B)**. Galectin-1 from astrocytes and galectin-3 from activated microglia promote neurite outgrowth and branching, by blocking Semaphorin 3A (Sema3A) pathway or binding to neural cell adhesion molecule L1 (NCAML1) and laminin, respectively **(A)**. Galectin-4 is segmentary localized to the axon by binding to sulfatide and NCAML1, regulating axonal growth and branching **(A)**. Oxidization and phosphorylation of galectin-1 and galectin-3, respectively, enhances neuroprotective activities of them **(A)**. In damaged neurons, galectin-3 from M1 type microglia enhances inflammation, whereas galectin-3 from M2 type microglia promotes phagocytic cleaning and oligodendrocyte differentiation **(B)**. Galectin-1, possibly from astrocytes, induces shift from the M1 to M2 phenotype of activated microglia and oligodendrocyte differentiation. Galectin-4 from regenerating axon inhibits maturation of pre-myelinating oligodendrocyte to determine the timing of (re)myelination.

Galectin-1,-3, and -4 are reported to be involved in the determination of axon fate, axon growth, guidance and regeneration, and polarized axonal glycoprotein transport (Figure 4A) (Higuero et al., 2017). Recombinant galectin-1 promotes neurite outgrowth of the cultured olfactory neuron (Puche et al., 1996). The oxidized form of galectin-1, which lacks lectin activity, stimulates neurite outgrowth (Outenreath and Jones, 1992; Horie et al., 1999; Inagaki et al., 2000), and there is convincing evidence showing that galectin-1 promotes neurite outgrowth and axonal regeneration in both the CNS and the PNS (Camby et al., 2006). Galectin-1 induces the repolymerization of F-actin in both growth cones and filopodium by inhibiting hydrogen oxide production and promoting axonal regeneration (Quintá et al., 2016). Similarly, microglial galectin-3 promotes neurite outgrowth in DRG explants (Pesheva et al., 1998), and phosphorylated galectin-3 promotes axon branching in cultured hippocampal neurons by local actin destabilization (Díez-Revuelta et al., 2010). Galectin-4 is sorted into discrete segments of the axonal membrane in a microtubule- and sulfatide-dependent manner and promotes axonal growth by clustering axon growth-promoting molecules, such as the neural cell adhesion molecule (NCAM) L1 (Velasco et al., 2013). Taken together, it is conceivable that galectin-1,-3, and -4 promote neurite outgrowth and branching, possibly binding to different partners, and post-translational modifications such as oxidization and phosphorylation are important for the neuroprotective function of galectins.

Galectin-1,-3, and -4 are also involved in (re)myelination in different ways (Figure 4B) (Rinaldi et al., 2016a; Thomas and Pasquini, 2018; de Jong et al., 2020). Galectin-3 secreted from M2 type activated microglia accelerates phagocytic cleaning of damaged cell debris and oligodendrocyte differentiation, whereas galectin-4 suppresses (re)myelination until appropriate axonal regeneration is completed (Figure 4B). Galectin-1, possibly secreted from astrocytes, promotes (re)myelination *via* shifting microglia phenotype from M1 to M2 (Figure 4B).

## Galectins and Neural Stem/Progenitor Cells

**Figure 5:**
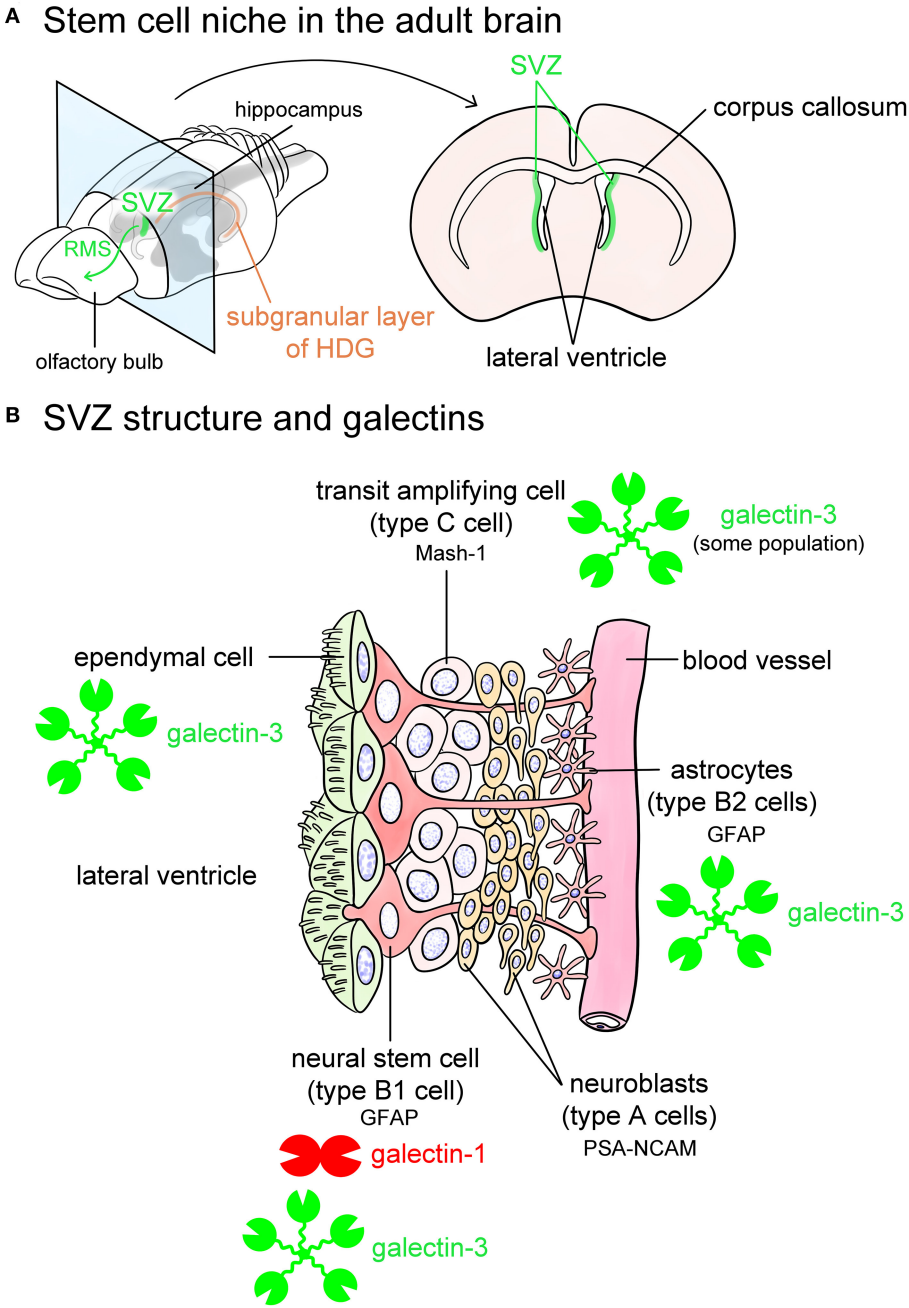
Galectins in the stem cell niches. Known stem cell niches in the adult brain are the subventricular zone (SVZ) (green in **A**) and subgranular layer of hippocampal dentate gyrus (HDG) (orange in **A**). The SVZ is located in the lateral wall of the lateral ventricles in the adult mouse brain **(A)**. Newly produced neurons in the SVZ migrate through rostral migratory stream (RMS) to the olfactory bulb (green arrow in **A**) at where they differentiate into interneurons such as periglomerular cells and granule cells, and also migrate to damaged lesions. The SVZ beneath ependymal cells is composed of glial fibrillary acidic protein (GFAP)-positive astrocytic neural stem cells (type B1 cells), astrocytes (type B2 cells) located along the blood vessels, mammalian achaete-scute homolog 1 (Mash-1)-positive transit amplifying cells (type C cells), and neuroblasts (type A cells) **(B)**. Type B1 neural stem cells express galectin-1, and possibly galectin-3. Some population of type C cells contains galectin-3 **(B)**. Galectin-3 is also abundant in ependymal cells and type B2 astrocytes **(B)**. Based on the researches by Sakaguchi et al. (2006), Comte et al. (2011), and Pasquini et al. (2011). PSA-NCAM, polysialylated form of neural cell adhesion molecule.

Recent studies indicate that the adult brain contains neural stem cells that continuously generate new neurons as well as glial cells, such as oligodendrocytes. Neurogenesis in the adult brain can be observed in the SVZ and the subgranular layer of the HDG (Figure 5A). The SVZ of the lateral wall of the lateral ventricles in adult mice is composed of astrocytic neural stem cells (type B1 cells), astrocytes (type B2 cells), transit amplifying cells (type C cells), and neuroblasts (type A cells) (Figure 5B) (Doetsch et al., 1997). Type B1 cells are neural stem cells that contain glial fibrillary acidic protein (GFAP), which differentiate into type C cells, followed by type A neuroblasts to generate new neurons (Figure 5B). Produced neurons migrate to the olfactory bulb through glial tubes formed by astrocytes, namely the rostral migratory stream (RMS), and they differentiate into interneurons such as periglomerular cells and granule cells in the olfactory bulb (Figure 5A). The SVZ also provides newly generated neurons to the damaged brain regions, such as infarct lesions induced by middle cerebral artery occlusion.

SVZ contains galectin-1 and -3 (Sakaguchi et al., 2006; Pasquini et al., 2011; Hillis et al., 2016; James et al., 2016). Galectin-1 is expressed in certain populations of GFAP-positive type B1 cells, namely neural stem cells in the SVZ (Sakaguchi et al., 2006). Comte et al. (2011) reported that galectin-3 is expressed in GFAP-positive astrocytes but not in neuroblasts in the SVZ. In contrast, Pasquini et al. (2011) insisted that galectin-3 immunoreactivity is negative in GFAP-positive type B1 neural stem cells. Galectin-3 is also abundant in RMS. Galectin-3 normally limits SVZ cell migration following cuprizone treatment (Hillis et al., 2016). Sirko et al. (2015) reported that both galectin-1 and -3 are increased in neural stem cells and reactive astrocytes in the lesioned brain following a stab wound injury. Neural stem cells in the HDG also contain galectin-1 and -3 (Kajitani et al., 2009; Imaizumi et al., 2011; Yang et al., 2012). Taken together, galectin-1 and -3 contribute to providing a stem cell niche and possibly play differential roles in a neural generation.

## Galectins in the CNS Under Pathological Conditions

Galectin-1 and 3 play important roles in the pathogenesis of neuroinflammatory and neurodegenerative disorders such as multiple sclerosis (MS), amyotrophic lateral sclerosis (ALS), Alzheimer's disease (AD), Parkinson's disease, Huntington's disease, and prion disease (Barake et al., 2020). Generally, galectin-1,-8, and -9 have anti-inflammatory and neuroprotective functions, but the role of galectin-3 is controversial. Galectin-3 facilitates pro-inflammatory action; however, it also plays an important role during the recovery period.

MS is a demyelinating neurodegenerative disease. Autoimmune T cells play an important role in the pathogenesis of MS. Several animal models for MS have been established, including experimental autoimmune encephalomyelitis (EAE), cuprizone or lysolecithin-induced demyelination models, and Theiler's murine encephalomyelitis virus (TMEV) infection model. The administration of recombinant galectin-1 improves both the clinical and histological scores of EAE (Offner et al., 1990). Galectin-1 has a neuroprotective role *via* anti-inflammatory action and regulates the microglial phenotype to stimulate re-myelination in EAE mouse model (Rinaldi et al., 2016b). On the contrary, the lack of galectin-3 improves EAE symptoms and reduces leukocyte infiltration, suggesting its pro-inflammatory function (Jiang et al., 2009). Galectin-3 may differentially contribute to the pathogenesis of EAE depending on cell type. In our experiment using a transfer EAE model in which autoimmune T cells were intravenously injected, both activated microglia and Schwann cells in the ventral but not dorsal roots become positive for galectin-3 whose expression patterns differ during progression and recovery periods: galectin-3 in phagocytic microglia elevates during symptom peaks while galectin-3 in Schwann cells appears during the recovery period (Itabashi et al., 2018). In a murine TMEV infection model, the loss of galectin-3 blocked an increase in chemokines after TMEV infection, reduced immune cell migration into the SVZ, reestablished SVZ proliferation, and increased the number of progenitors in the corpus callosum (James et al., 2016). The loss of galectin-8 increases T helper (Th)17 cells but decreases T regulatory cells, suggesting that galectin-8 eliminates activated Th17, but not Th1, and ameliorates EAE (Pardo et al., 2017). TNFα increases galectin-9 expression in astrocytes, inducing apoptosis of encephalitogenic T-cells (Steelman et al., 2013). Increased expression of galectin-1,-3,-8, and -9 has been confirmed in human MS lesions (Stancic et al., 2011), and the concentration of anti-galectin-1 autoantibodies is increased in the serum of MS patients (Lutomski et al., 1997). The cerebrospinal fluid of MS patients contains the anti-galectin-8 antibody, and its increase is related to relapse (Pardo et al., 2017).

ALS is a progressive neurological disorder in which motor neurons are selectively impaired in the brain and spinal cord. Galectin-1,-3, and -9 are increased in SOD1^G93A^ mice, an animal model of ALS and the spinal cord, cerebrospinal fluids, and serum of ALS patients (Zhou et al., 2010; Lerman et al., 2012; Peters et al., 2015; Ashraf and Baeesa, 2018). Galectin-1 is found in a component of the neurofilamentous lesions in ALS patients (Kato et al., 2001) and localizes to swollen motor axons and astrocytes in SOD1^G93A^ mice (Kobayakawa et al., 2015). Chang-Hong et al. (2005) reported that oxidized galectin-1 improves the clinical symptoms of SOD1^G93A^ mice, suggesting its neuroprotective role. Galectin-3 is increased in microglia in the spinal cord of ALS patients and phagocytosing damaged γ-synuclein-positive neurons (Peters et al., 2015). Galectin-3-knockout mice have severe clinical significance, suggesting a protective role of galectin-3 in neural damage in ALS (Lerman et al., 2012).

AD is caused by abnormal accumulation of Aβ and tau proteins in the brain, damaging neurons and affecting gradual brain degeneration and atrophy. Galectin-3 is highly upregulated in microglia associated with Aβ plaques in the brains of AD patients and the familial AD model, 5xFAD mice (Boza-Serrano et al., 2019). Since single-nucleotide polymorphisms in the galectin-3-encoding *LGALS3* gene being associated with an increased risk of AD and galectin-3 deletion decreasing Aβ burden in 5xFAD mice, whereas injected galectin-3 promotes aggregation of insoluble Aβ, galectin-3 may accelerate AD progression (Boza-Serrano et al., 2019). In Aβ-induced neuroinflammation in the hippocampus, galectin-3 expression is increased in microglia and astrocytes (Ramírez et al., 2019). In an animal model of AD, APP/PS1 mice, galectin-3 overexpression enhanced the accumulation of Aβ oligomerization (Tao et al., 2020). In mice lacking galectin-3, the Aβ-degrading enzyme neprilysin is increased, showing better acquisition and retention performance in the water maze (Tao et al., 2020). Galectin-3 is reported to be increased in the frontal lobe in association with Aβ oligomerization (Tao et al., 2020) and in the serum of AD patients (Wang et al., 2015). Galectin-8 regulates autophagy by binding to NDP52 (Thurston et al., 2012), and inhibition of galectin-8 promotes tau aggregation in cultured cells (Falcon et al., 2018).

Few reports show the involvement of galectins in Parkinson's and Huntington's diseases, which are caused by a loss of dopamine neurons in the substantia nigra, inducing progressive movement disorders such as tremor, stiff, and inflexible muscles. Galectin-1 plays an anti-inflammatory function and suppresses neuronal degeneration in the 1-methyl-4-phenyl-1,2,3,6-tetrahydropyridine-induced Parkinson's disease model (Li et al., 2020). Galectin-3 is involved in microglial activation by accumulating α-synuclein, which induces the loss of dopamine neurons (Boza-Serrano et al., 2014). The serum concentrations of galectin-3 and -4 are increased, but galectin-1 in the cerebrospinal fluids is decreased in patients with Parkinson's disease (Cengiz et al., 2019; Marques et al., 2019; Yazar et al., 2019). Galectin-3 is also increased in the serum of patients with Huntington's disease (Siew et al., 2019).

Prion diseases are infectious and fatal neurodegenerative diseases, including scrapie in sheep and goats, bovine spongiform encephalopathy in cows, and Creutzfeldt-Jakob disease in humans. Prion diseases are caused by the accumulation of PrP^Sc^, which is an abnormal and relatively proteinase K-resistant isoform of cellular prion protein PrP^C^, inducing gradual neuron loss and astrocytosis, inducing transmissible spongiform encephalopathies. Galectin-3 is identified as one of the upregulated genes in prion disease (Riemer et al., 2004), which increased in activated microglia in the brains of scrapie-infected mice, contributing to disease development by regulating lysosomal function and autophagy (Jin et al., 2007; Mok et al., 2007). Galectin-1, especially the S-nitrosylated form of galectin-1, is upregulated in the brains of scrapie-infected rodents and human prion diseases, where astrocytes and neurons, but not microglia, are immunoreactive for galectin-1 (Guo et al., 2017).

Galectin-1 and -3 knockout mice exhibit impaired stress-coping and increased compulsive-like behavior, implying that galectin-1 and -3 are related to depression and obsessive-compulsive-like behaviors (Sartim et al., 2020). Serum galectin-3 level is elevated in association with higher levels of depressive symptoms (King et al., 2021). Recent studies noted an involvement of galectins, especially galectin-3 and -8 in schizophrenia (Kajitani et al., 2017; Petralia et al., 2021). Although information about galectins in mental illness such as depression, schizophrenia, and autism is limited, galectins are likely involved in etiology of these disease.

## Ligands for Galectins in the CNS

Several ligand glycoconjugates for galectins in the CNS have been identified and summarized in Table 1. They include molecules that regulate neurite outgrowth and branching, myelination, cell-extracellular matrix adhesion, and inflammatory responses.

**Table 1:** Ligands for galectins in the CNS.

**Galectin**	**Ligand glycoconjugates**	**Region/cell**	**Function**	**References**
Galectin-1	Lactosamine epitopes in glycolipids (recognized by mAb 1B2) Laminin family members	Olfactory nerve	Olfactory axon fasciculation	Mahanthappa et al., 1994
	β1 integrin	Neural progenitor cells in SVZ	Regulate the number of neural stem cells	Sakaguchi et al., 2010
	CD45 (core 2 *O*-glycan)	M1 microglia	M1 microglia de-activation	Starossom et al., 2012
	Neuropilin-1/plexinA4 receptor complex	Injured neuron	Axonal regeneration, locomotor recovery	Quintá et al., 2014
Galectin-3	Laminin	DRG neurons, PC12 cells (pheochromocytoma)	Cell adhesion and neurite outgrowth	Pesheva et al., 1998
	NCAML1 (Neural cell adhesion molecule L1)^a^	Cultured hippocampal neurons	Axon branching	Díez-Revuelta et al., 2010
	Toll-like receptor 4 (TLR4)	Microglia	Sustained microglia activation	Burguillos et al., 2015
	Triggering receptor on myeloid cells 2 (TREM2)	Microglia (5xFAD AD model mice)	Pro-inflammatory cytokine production	Boza-Serrano et al., 2019
	Bone morphogenetic protein receptor 1α (BMPR1α)	SVZ	Astrogenesis	Al-Dalahmah et al., 2020
	Amyroid Aβ monomer	APP/PS1 mice (AD model)	Oligomerization of Aβ, decreasing degradation of Aβ	Tao et al., 2020
Galectin-4	p27	Oligodendrocytes	Oligodendrocyte maturation	Wei et al., 2007
	Sp1			
	Suflatide NCAML1	Hippocampal and cortical neurons	Axonal elongation	Velasco et al., 2013
	Galactosyl ceramide Sulfatide	Immature oligodendrocytes	Inhibit myelination	Stancic et al., 2012
Galectin-8	α3, α5, β1 integrins	Hippocampal neurons	Protect neurons from harmful conditions	Pardo et al., 2019
Galectin-9	T cell immunoglobulin and mucin domain 3 (Tim3)	Encephalitogenic T cell (EAE model)	Apoptosis of encephalitogenic T cells	Steelman et al., 2013

a *Molecules to which phosphorylated form of galectin-3 binds*.

*AD, Alzheimer's disease; DRG, dorsal root ganglion; EAE, experimental autoimmune encephalomyelitis; mAb, monoclonal antibody; SVZ, subventricular zone*.

## Discussion

Galectins are distributed both in neurons and glial cells, regulating their growth, differentiation, and function in different ways. They are also involved in the pathogenesis of neuroinflammatory and neurodegenerative diseases in the CNS. Generally, galectin-1,-8, and -9 act as neuroprotective and anti-inflammatory agents, whereas galectin-3 has multiple functions depending on the cell type and context. Galectin-3 is upregulated in activated microglia in both M1 and M2 phenotypes. Galectin-3 in M1 type microglia acts as pro-inflammatory, whereas galectin-3 in M2 type microglia facilitates phagocytic activity and neuroprotective and anti-inflammatory functions. Thus, the action of galectin-3 is often controversial, depending on disease status. Although we still do not know what determines the differential activity of galectin-3 on microglial function, it may be due to the differential glycoconjugate expression to which galectin-3 binds in different types of microglia or surrounding environmental cells and extracellular matrix. Galectin-3 may act as pattern recognition receptors (PRRs) such as TLRs that recognize pathogen-associated molecular pattern molecules (PAMPs) such as lipopolysaccharides (LPS) and damage-associated molecular pattern molecules (DAMPs) such as high-mobility group box-1 in innate immunity. Galectin-3 and -9 also act as DAMPs, and galectin-3 can bind to TLR4, participating in initial neuroinflammatory processes through secretion of pro-inflammatory cytokines such as TNFα, IL-1β, and IL-6 (Burguillos et al., 2015; Rahimian et al., 2021). Galectin-3 controls the severity of inflammation and induces the activation of NLRP3 inflammasome-dependent pathways in Huntington's disease and spinal cord injuries (Ren et al., 2019; Siew et al., 2019). Galectin-3 may also play a role in inflammation amplifiers in neurodegenerative diseases (Rabinovich et al., 2002) (Figure 4B). In contrast, galectin-3 influences microglial polarization to the M2 phenotype through the IL-4 receptor pathway, possibly by binding to the glycan on the IL-4 receptor (Rahimian et al., 2019, 2021). Although the underlying mechanism to define the function of galectin-3 in neurodegenerative disease is not fully understood, it may depend on the timing and context of disease status.

Post-translational modification of galectins influences the localization and function of galectins, especially galectin-1 and -3 (Gao et al., 2017). Galectin-3 consists of a *C*-terminal CRD and *N*-terminal peptide, required for oligomerization (Figure 1B). *N*-terminal peptides contain serine and tyrosine residues that are phosphorylated by kinases, such as casein kinase I, and the sites cleaved by several proteinases, such as MMPs. Galectin-3 usually exists as a monomer but can also oligomerize *via* self-association of the *N*-terminal domain to form pentamers in the presence of multivalent oligosaccharides containing terminal LacNAc. Galectin-3 crosslinks cell surface glycoproteins and extracellular matrix to mediate cell-to-cell and cell-to-matrix adhesion, as well as the trafficking and presentation of surface glycoproteins. Cleavage of the *N*-terminal domain by proteases results in the abrogation forming oligomer, whereas released CRD has a high glycan-binding affinity (Ochieng et al., 1994). Because galectin-3 appears to be cleaved in OPCs and OPCs contain a plant lectin PNA-positive glycans as the substrate to generate core 2 type *O*-glycans, in which elongation of poly-LacNAc is formed (Figure 1C) (Pasquini et al., 2011), galectin-3-glycan-binding seems to be enhanced in OPC without forming lattice on cellular membrane. In contrast, serine phosphorylation by casein kinase I reduces the glycan-binding activity of galectin-3, while it regulates the galectin-3 cellular distribution, which is important for its anti-apoptosis/anoikis activity (Gao et al., 2017). As mentioned above, phosphorylated galectin-3 promotes axon branching in cultured neurons (Díez-Revuelta et al., 2010). Oxidization, biologically induced by reactive oxygen species such as hydrogen peroxide, attenuates the glycan-binding ability of galectin-1. The promoting action of galectin-1 on neurite outgrowth is not dependent on its lectin properties (Inagaki et al., 2000). Tamura et al. (2015) reported that nitric oxide-caused S-nitrosylation of cysteine residues in CRD of galectin-2, which is intensely expressed in the stomach and small intestine, prevents the hydrogen peroxide-induced inactivation of glycan-binding activity. In prion diseases, S-nitrosylated galectin-1 is increased in scrapie-infected rodents and human prion diseases, where neuroprotection of galectin-1 does not appear to function efficiently (Guo et al., 2017). Since the oxidized form of galectin-1 lacking lectin activity promotes axonal regeneration at much lower concentrations at which galectin-1 exhibits lectin activity (Inagaki et al., 2000), S-nitrosylation of galectin-1, which protects against oxidization to maintain lectin activity, may attenuate its neuroprotective action in prion disease. Thus, local environment influences the glycan-binding ability of galectins and may change their functions.

As mentioned in the introduction, the affinity of galectins for glycan is enhanced by increased branching and repeated LacNAc motifs. Branching of complex type *N*-glycans is mediated by mannose *N*-acetylglucosaminyl transferases (Mgat1, 2, 4, 5/GnTI, II, IV, V) that transfer GlcNAc to core trimannoses on *N*-glycans *via* α1,3 or α1,6 linkages to form branches (Figure 1C). Mgat5/GnTV promotes fourth branching, where galectins, especially galectin-3, form galectin lattice by binding to poly-LacNAc units, regulating trafficking and presentation of cell surface proteins. Prof. James W. Dennis and Prof. Michael Demetriou have extensively investigated the immunosuppressive effect of Mgat5-mediated *N*-glycan branching and the formation of the galectin lattice (Demetriou et al., 2001). Differentiation of peripheral T cells into pro-inflammatory Th1 and Th17 cells is inhibited by the galectin lattice, which combines with T cell receptor regulation, and determines the risk of autoimmune diseases such as MS (Demetriou et al., 2001; Lee et al., 2007; Grigorian et al., 2011). In addition to immune regulation, the galectin lattice drives myelination by promoting the surface retention of PDGFRα in OPC, a receptor that plays a critical role in oligodendrogenesis (Sy et al., 2020). Mgat5-mediated branching and poly-LacNAc formation depend on GlcNAc availability in cells. Since oral administration of GlcNAc drives anti-inflammatory action as well as myelin repair, reducing axonal damage and enhancing functional recovery in EAE mice (Grigorian et al., 2011; Lee et al., 2019), GlcNAc is a new therapeutic supplement for MS patients. Oral GlcNAc administration in patients with MS is under clinical trial led by Dr. M. Demetriou's group.

It is well known that glycosaminoglycans such as chondroitin sulfate and keratan sulfates inhibit neurite outgrowth (Snow et al., 1990; Cole and McCabe, 1991), and chondroitinase ABC is a promising therapeutic agent for CNS regeneration. Keratan sulfate is abundantly located in the cartilage, cornea, and the brain, and contains a repeated LacNAc units (Figure 1C). Iwaki et al. (2008) demonstrated that de-sulfated keratan sulfates, as well as de-sulfated chondroitin/dermatan sulfates, are potential galectin ligands, especially galectin-3 and -9. Sulfation and sialylation on keratan sulfate in microglia influence the progression and recovery of spinal cord injuries, ALS, and AD (Ito et al., 2010; Hirano et al., 2013; Foyez et al., 2015; Ueno et al., 2015; Zhang et al., 2017). Deficiency of arylsulfatase A and B, that are associated with metachromatic leukodystrophy and Maroteaux-Lamy syndrome, respectively, increases sulfatides and sulfated chondroitin 4-sulfate in the CNS, attenuating oligodendrocyte differentiation and neural recovery (Zhang et al., 2014; Bhattacharyya et al., 2015; Pituch et al., 2015). Increased sulfation in glycosaminoglycans as well as glycolipids likely impairs glycan-binding of galectin-3, resulting in reduced its functions. Sulfotransferase and sulfatase activity may link to the galectin-3 action during neuroinflammation and neural regeneration. Because GlcNAc treatment enhances the galectin-3-glycan-binding *via* increasing *N*-glycan branching and improve (re)myelination by forming lattice on PGDFRα as mentioned above, it also would be a potential therapeutic strategy for genetic disorders such as metachromatic leukodystrophy with increased sulfation in glycosaminoglycans and glycolipids.

Because the availability of glucose and glutamine is related to *de novo* synthesis of UDP-GlcNAc required for *N*-glycan branching, thus influencing galectin lattice formation, there may be a link between energy conditions and galectin expression and glycan composition in each cell (Araujo et al., 2017). During ischemia, the brain is exposed to hypoglycemic and hypoxic conditions. In the hypoxic tumor microenvironment, inhibition of α3β1 integrin translocation to the plasma membrane, decreased 1,2-fucosylation of cell-surface glycans, and galectin overexpression was consequently observed by alteration of intracellular glucose metabolism from aerobic cellular respiration to anaerobic glycolysis (Silva-Filho et al., 2017). Although the mechanisms driving the change in glycosylation status and galectin expression are still unclear, the glucose availability and metabolism pathway are conceivable to be an essential key to elucidating the interactive changes in glycans and galectins. Although evidence showing the relationship between galectin expression and the glucose metabolism pathway, which leads to changes in glycan structure in cells, is limited, to revealing their connection will provide beneficial information to elucidate the function of galectins and the ligand glycoconjugates in the regulation of physiological and pathological events in the CNS.

Ligand glycoconjugates for galectins serve not only glycoproteins but also glycolipids as well as extracellular proteoglycans. Multiple galectins are involved in the regulation of physiological and pathological events in the CNS in different ways. In addition to galectins, other lectins, such as sialic acid-recognizing siglecs, are also involved in the pathogenesis of neurodegenerative diseases (Siddiqui et al., 2019; Puigdellívol et al., 2020). Therefore, it is necessary to understand the overall regulation of glycans and lectins. To find common or individual rules regulating cellular glycan-lectin properties will be beneficial to reveal the etiology and pathogenesis of neurodegenerative diseases and to establish new therapeutic strategies.

## Author Contributions

All authors listed have made a substantial, direct and intellectual contribution to the work, and approved it for publication.

## Funding

This work was funded by the Medtronic Japan External Research Institute Foundation.

## Conflict of Interest

The authors declare that the research was conducted in the absence of any commercial or financial relationships that could be construed as a potential conflict of interest.

## Publisher's Note

All claims expressed in this article are solely those of the authors and do not necessarily represent those of their affiliated organizations, or those of the publisher, the editors and the reviewers. Any product that may be evaluated in this article, or claim that may be made by its manufacturer, is not guaranteed or endorsed by the publisher.
